# *Cryptosporidium parvum* vaccine candidates are incompletely modified with *O*-linked-*N*-acetylgalactosamine or contain N-terminal *N*-myristate and *S*-palmitate

**DOI:** 10.1371/journal.pone.0182395

**Published:** 2017-08-08

**Authors:** John R. Haserick, Joshua A. Klein, Catherine E. Costello, John Samuelson

**Affiliations:** 1 Department of Molecular and Cell Biology, Boston University Goldman School of Dental Medicine, Boston, Massachusetts, United States of America; 2 Department of Biochemistry, Boston University School of Medicine, Boston, Massachusetts, United States of America; 3 Program for Bioinformatics, Boston University, Boston, Massachusetts, United States of America; University of Georgia, UNITED STATES

## Abstract

*Cryptosporidium parvum* (studied here) and *Cryptosporidium hominis* are important causes of diarrhea in infants and immunosuppressed persons. *C*. *parvum* vaccine candidates, which are on the surface of sporozoites, include glycoproteins with Ser- and Thr-rich domains (Gp15, Gp40, and Gp900) and a low complexity, acidic protein (Cp23). Here we used mass spectrometry to determine that *O*-linked GalNAc is present in dense arrays on a glycopeptide with consecutive Ser derived from Gp40 and on glycopeptides with consecutive Thr derived from Gp20, a novel *C*. *parvum* glycoprotein with a formula weight of ~20 kDa. In contrast, the occupied Ser or Thr residues in glycopeptides from Gp15 and Gp900 are isolated from one another. Gly at the N-terminus of Cp23 is *N*-myristoylated, while Cys, the second amino acid, is *S*-palmitoylated. In summary, *C*. *parvum O*-GalNAc transferases, which are homologs of host enzymes, densely modify arrays of Ser or Thr, as well as isolated Ser and Thr residues on *C*. *parvum* vaccine candidates. The N-terminus of an immunodominant antigen has lipid modifications similar to those of host cells and other apicomplexan parasites. Mass spectrometric demonstration here of glycopeptides with *O*-glycans complements previous identification *C*. *parvum O*-GalNAc transferases, lectin binding to vaccine candidates, and human and mouse antibodies binding to glycopeptides. The significance of these post-translational modifications is discussed with regards to the function of these proteins and the design of serological tests and vaccines.

## Introduction

*C*. *parvum* infects humans and cows, while *C*. *hominis* only infects humans [1–3]. *C*. *parvum* was first identified as an opportunistic pathogen and cause of severe diarrhea in AIDS patients [4, 5]. In 1993, *C*. *parvum* contaminated the municipal water supply in Milwaukee, Wisconsin, U.S.A and caused a massive outbreak of diarrhea among immunocompetent persons [6, 7]. More recently *C*. *parvum* has been shown to be the second most important cause (after rotavirus) of diarrhea and death in infants in low resource countries where the parasite is endemic [8–10]. Presently, there are no human vaccines for *C*. *parvum*, although numerous candidates have been identified [11, 12]. Treatment of *C*. *parvum* is difficult in populations with the most severe disease: infants and immunosuppressed persons [1, 13].

Oocysts of *C*. *parvum* have acid-fast (lipid-rich) walls, which are resistant to environmental insults and to gastrointestinal acids, proteases, and bile [5, 14]. Oocysts each contain four infectious sporozoites, which have on their surface Ser- and Thr-rich glycoproteins (*e*.*g*. Gp900 and Gp40) [15–20]. The precursor protein (Gp40/Gp15), which is specific for *C*. *parvum* and *C*. *hominis*, is cleaved by a furin-like protease into an N-segment (Gp40) and a C-segment (Gp15) [21]. Subsequently, the N-terminal signal peptide of Gp40 is removed, and a glycosylphosphatidylinositol (GPI) anchor is added to the C-terminus of Gp15 [22]. Gp40 contains a domain of 17 consecutive Ser residues followed by Thr-Ser-Thr, while the Ser and Thr residues of Gp15 are dispersed. Gp900, although much larger than Gp40/Gp15, is a secreted protein present in *C*. *parvum*, *C*. *hominis*, and *C*. *muris*. Gp900 has four sets of consecutive Thr residues, ranging in length from 33 to 155 residues, as well as dispersed Ser and Thr [16, 17]. Gp900, which is shed from the surface of sporozoites during gliding motility, tethers sporozoites to the interior surface of the oocyst wall [15, 16, 23]. In contrast, Gp15 is present at the apical end of sporozoites and on the outer surface of the oocyst wall [15]. Sporozoites also make a galactose/GalNAc-specific lectin, which interacts with Gp900 and Gp40 [24].

Polymorphisms in Gp15 and Gp40 have been used to distinguish among isolates of *C*. *parvum*, while recombinant Gp15 has been used to measure the serological response in epidemiological studies [25–30]. Vaccination studies have been performed using recombinant *C*. *parvum* proteins, bacterial vectors (*e*.*g*. *Salmonella*), or DNA encoding *C*. *parvum* proteins [11, 12, 31]. These vaccines either contain no *O*-glycans (bacterially expressed proteins) or may display host *O*-glycans (DNA vaccines). The presence of *O*-glycans (most likely *O*-GalNAc) on *C*. *parvum* glycoproteins has not previously been detected by mass spectrometry, but it has been suggested on the basis of the following five observations: (1) The *C*. *parvum* genome predicts four *O*-GalNAc transferases (*O*-GalNAcTs), and parasite lysates add *O*-GalNAc to synthetic peptides [32]. (2) A lectin that recognizes *O*-GalNAc (*Helix pomatia* agglutinin) (HPA) binds to the surface of sporozoites, while binding of a monoclonal antibody (4E9) to Western blots of *C*. *parvum* proteins is competed by HPA and reduced by treating proteins with an *O*-GalNAcase [25]. (3) The *Maclura pomiphera* agglutinin, which binds *O*-GalNAc, dramatically enriches Gp40, Gp900, and other mucin-like glycoproteins of *C*. *parvum* [15]. (4) Sera from patients infected with *C*. *parvum* bind to synthetic peptides containing *O*-linked GalNAc [33]. (5) *O*-GalNAc is added to *C*. *parvum* Gp40 exogenously expressed in *Toxoplasma gondii* [34].

Cp23, which is also known as the immunodominant antigen, is a small, low complexity, acidic protein of sporozoites [35]. Monoclonal antibodies to Cp23 partially protect neonatal mice against oral infection with *C*. *parvum*, while antibodies to Cp23 have more frequently been found in HIV/AIDS patients infected with *C*. *parvum* but without diarrhea [36, 37]. Recombinant Cp23 has been used to demonstrate humoral and cellular immune responses to *C*. *parvum* in human, cattle, and mouse infections, whereas recombinant Cp23 and DNA-based vaccines have been used to immunize mice and to elicit an innate immune response from mouse and human dendritic cells *in vitro* [29, 38–45]. Mass spectrometric studies of *C*. *parvum* sporozoites and oocysts have identified numerous peptides from Gp40, Gp15, Gp900, and Cp23, but none of these studies localized post-translational modifications (PTMs), which may include *O*-linked glycans, Asn-linked glycans (*N*-glycans), and fatty acyl chains [15, 22, 32, 33, 46–51]. Recently we used mass spectrometry to determine that *C*. *parvum N*-glycans, which are built on a predicted precursor with a single long mannose arm, appear to be processed by glucosidase-2 but not by ER mannosidases, and are not modified by Golgi glycosyltransferases [52–54]. The resulting *N*-glycans, which are likely GlcMan_5_GlcNAc_2_ and Man_5_GlcNAc_2_, are remarkable for their simplicity, as compared to the complicated *N*-glycans identified in other protists [55]. In this report, we used mass spectrometry to characterize tryptic glycopeptides of lysates of *C*. *parvum* oocysts and thereby directly determine the number and some of the positions of *O*-GalNAc residues on Gp40, Gp15, Gp900, and a previously uncharacterized glycoprotein with a predicted weight of 20-kDa (named here Gp20). Mass spectrometry of hydrophobic peptides also detected the addition of myristoyl and palmitoyl groups to the first and second residues, respectively, at the N-terminus of Cp23 [56, 57].

## Materials and methods

### Reagents and parasites

Freshly passaged *C*. *parvum* oocysts were purchased from Bunch Grass Farm (Deary, ID) and handled under BSL-2 protocols, with the approval of the Boston University Institutional Biosafety Committee. All reagents and chemicals were purchased from Sigma-Aldrich (St. Louis, MO), unless noted otherwise. Solvents used for LC-MS were Optima™ grade, procured from Fisher Scientific (Thermo-Fisher Scientific, Waltham, MA).

### Protein extraction and trypsin digestion

Procedures for extracting proteins from *C*. *parvum* oocysts and digesting them with trypsin have recently been described in detail [54], and so a summary of the methods is presented here. Briefly, 10^9^ *C*. *parvum* oocysts were concentrated by centrifugation, washed 3X with PBS, and resuspended with PBS containing EDTA-free cOmplete^TM^ protease inhibitor (Roche, Basel, Switzerland). Oocyst walls were disrupted in a bead beater with 0.5-mm glass beads and centrifuged. The PBS supernatant was removed and saved, while the remaining insoluble materials and beads were extracted with a solution composed of 10 mM HEPES, 25 mM KCl, 1 mM CaCl_2_, 10 mM MgCl_2_, 2% CHAPS, 6 M guanidine HCl, 50 mM dithiothreitol, 1X protease inhibitor, pH 7.4. The resulting guanidine-DTT supernatant was combined with the PBS supernatant, and the insoluble material was discarded. The proteins were then precipitated, and the pellet was washed with methanol and vacuum dried. Alternatively, oocyst proteins were extracted with hot phenol, and phenol and interphase layers were kept, while the aqueous layer was discarded. Proteins were precipitated with methanol containing 100 mM NH_4_OAc and dried. The pelleted proteins were resuspended in 50 mM NH_4_HCO_3_, pH 8.0, reduced with 50 mM DTT, alkylated with iodoacetamide, and then digested with proteomics grade trypsin (Sigma-Aldrich, St. Louis, MO). Tryptic peptides were dried and desalted using C18 ZipTip concentrators following the manufacturer’s protocol (EMD Millipore, Danvers, MA).

### Mass spectrometry

The LC-MS/MS methodologies and the manual interpretation of MS/MS spectra of *C*. *parvum* glycopeptides containing *O*-glycans were performed using the methods described for *C*. *parvum N*-glycosylated peptides [54]. Desalted and dried peptides from three biological replicates were dissolved in 2% ACN, 0.1% formic acid (FA) and separated using a NanoAcquity Ultra Performance Liquid Chromatography (UPLC) system (Waters. Milford, MA), fitted with a nanoAcquity Symmetry C18 trap column and a BEH130C18 analytical column. Solvent mixtures for the mobile phase gradient were 99:1:0.1 HPLC grade water/ACN/FA and 99:1:0.1 ACN/HPLC grade water/FA. The UPLC was coupled to a TriVersa NanoMate ion source (Advion, Ithaca, NY), operated at 1.5 kV to introduce ions into either an LTQ-Orbitrap-XL or a QE Plus mass spectrometer (Thermo-Fisher Scientific, San Jose, CA). Both mass spectrometers were operated in the positive-ion mode. MS^1^ spectra were recorded over the range *m/z* 350–2000. MS^2^ HCD spectra were acquired by isolating the top 5 (LTQ-Orbitrap) or top 20 (QE+) precursor ions with a 2-*m/z* window and fragmenting the selected precursor ions with 15–45 V HCD energy. The lower energy MS^2^ HCD spectra were scanned from *m/z* 100 to an upper *m/z* value, which was dependent upon the parent ion *m/z*. For the 45-V HCD spectra, ions below *m/z* 210 were excluded to avoid trapping the very abundant HexNAc oxonium ion.

#### Manual interpretation of mass spectra

Data obtained from LC-MS/MS experiments were first examined using Qual Browser in the Xcalibur 2.2 software suite (Thermo-Fisher Scientific). Extracted ion chromatograms were generated from MS/MS spectra for oxonium ions of interest (HexNAc, *m/z* 204.0866; Hex-HexNAc *m/z* 366.1395; HexNAc_2,_ *m/z* 407.1670). Spectra containing one or more of these ion(s) were then manually interpreted [54]. Once a sequence was obtained, it was searched against the 3,803 entries within the *C*. *parvum* Iowa-II predicted proteome and cross-searched within the entire NCBI nr database, using the online NCBI BLASTP algorithm (https://blast.ncbi.nlm.nih.gov/Blast.cgi) [58–60]. The software, Glycoworkbench v2.1, release 146, was used to help calculate glycan compositions [61]. *O*-glycosylated peptides utilized HexNAc almost exclusively. Due to the labile nature of *O*-linked glycans, b and y ions containing one or more HexNAc residues typically had very low abundances. The charge-reduced molecular ion that had undergone the loss of one or more HexNAc residues was often observed. The information obtained from manual interpretations was then used for database searches, allowing for deeper sequencing of the data and higher throughput processing of samples. The peak list, the assigned ions, and their mass errors for manually annotated spectra shown in the figures are listed in (S2 Excel File).

#### Database searches for glycopeptides

Automated database searches were performed using the PEAKS software suite version 8.0 (Bioinformatics Solutions Inc., Waterloo, ON, Canada), using recently described methods for *N*-glycans with modifications [54]. The search criteria were set as follows: trypsin as the enzyme with ≤ two missed cleavages and ≤ one non-specific cleavage, the error tolerances for the precursor of 6 ppm and 0.02 Da for fragment ions, carbamidomethyl cysteine as a fixed modification, and the dynamic modifications on Ser/Thr with (HexNAc to HexNAc_4_) with ≤ six/peptide. The peptide match threshold (-10 logP) was set to 15, with estimation of the false discovery rate (FDR), a 5.6 FDR was calculated. A multi-round search was performed using the *de novo* only results from the first PEAKSDB search to find peptides with attached lipids. The second search parameters were identical to the prior PEAKSDB search, with the exception that myristate (N-term, Ser, Thr) and palmitate (Cys, Ser, Thr, Lys) were specified as dynamic modifications, and HexNAc modifications were removed. The results from the searches were exported into Excel and collated.

#### Re-annotation of glycopeptides from automated database searches

For multiple reasons, the PEAKS DB search algorithm failed to annotate the product ions appropriately. Therefore, the glycopeptide results from the PEAKSDB search were exported in mzIdentML 1.1 format [62], manually verified, then provided to GlycReSoft (a software package developed in-house for glycopeptide discovery and annotation). The code for GlycReSoft, which is currently in active development with periodic updates and improvements, is open source and freely available from the online repository: https://github.com/BostonUniversityCBMS/glycresoft. All peptides listed in the mzIdentML document and all non-redundant theoretical tryptic digest peptides for each included protein were used as templates, upon which a database of theoretical glycopeptides was constructed. Glycosylation was permitted at up to 20 putative sites. All distinct combinations-with-replacement with the putative glycan compositions were generated. For each template peptide, theoretical glycopeptides were produced by assigning glycosylation events for combinations of between 1 and *k* glycosylation sites, where *k* is the total number of potential glycosylation sites. The combinatorial complexity was reduced by limiting the number of possibilities to the first 100 combinations, for glycopeptides having an excess of 100 possible placements.

Each dataset was deisotoped, charge state deconvolved, and searched independently against the database described above. Individual datasets of MS/MS scans in the range m/z 100–240 were filtered, and only tandem mass spectra for which the average ratio of oxonium ion signal to maximum signal exceeded 5% were considered. In addition to including normal peptide backbone fragments, the search considered spectra containing peaks that indicated either the presence of a HexNAc residue or its loss. The software also searched for the intact peptide backbone with zero or more partial losses of each potential glycan. Glycopeptide-spectrum matches were evaluated based upon joint binomial intensity-backbone coverage criteria, which included in a novel algorithm that is based in part on a binomial scoring function described previously [63]. The lists of ions assigned for each of these spectra are located in S1 Excel File. S4 Fig shows a representative spectrum annotated by GlycReSoft (one of 345) submitted to the ProteomeXchange Consortium [64].

#### Other bioinformatic methods

The furin-like protease site that separates Gp40/Gp15 was predicted by the online tool “ProP 1.0 Server”, made available by the Center for Biological Sequence Analysis, Department of Systems Biology, Technical University of Denmark (www.cbs.dtu.dk/services/ProP/) [65]. Signal peptides and transmembrane helices were predicted using the online tool Phobius (http://phobius.sbc.su.se/index.html) [66]. The GPI-anchor site of Gp15 was predicted using the BIG-PI prediction server (http://mendel.imp.ac.at/gpi/gpi_server.html) [67]. Cartoon representations of proteins and protein features, which were mapped with all the peptides across all MS/MS experiments, were generated using the online software tool Protter v.1.0 (http://wlab.ethz.ch/protter/start/) [68]. The assigned peptides from the PEAKSDB search results were used to map to the proteins of interest. The protein features were mapped using the results from the bioinformatics searches.

### *O*-linked glycan release and characterization

Ser-linked or Thr-linked glycans were released from the proteins via reductive alkaline β-elimination. Briefly, purified proteins from a total oocyst lysate were first lyophilized in glass conical vials. To the dried protein extract, an aqueous solution of 0.1 M NaOH + 1 M NaBD_4_ was added. The loosely capped vials were placed into an oven and kept at 45°C for 18 h. After the incubation period, the borate was removed by extensive washes with 10% acetic acid in methanol and then neat methanol. The released glycans were subsequently separated from the proteins by solid phase extraction columns. To the dried sample, LC-MS grade water containing 0.1% trifluoroacetic acid (TFA) was added; the tube was vigorously vortexed, and the contents were then passed through a C-18 Sep-Pak cartridge (Waters Corporation, Milford, MA). Three bed volumes of 0.1% TFA/water were subsequently passed through the column, and the eluent fractions were pooled and lyophilized. The released *O*-glycans were permethylated using previously described methods [69, 70]. A slurry of powdered NaOH in DMSO was added to the dried, released *O*-glycans. An equal volume of methyl iodide was added, and the reaction mixture was agitated gently while protected from light. The process was repeated three times to ensure complete permethylation. The product was extracted with chloroform/water, and the aqueous layer was removed and discarded. Washes with water were repeated until the pH of the solution was that of the LC-MS grade water being used for the washes. The chloroform layer was removed, placed into a new clean vial, dried in a speed vacuum and stored in a dissector at -20°C until it was analyzed.

#### Monosaccharide composition determination by GC-MS

The permethylated sugars were identified using GC-MS with a Bruker Scion SQ interfaced to a 436-GC (Bruker Daltonics, Billerica, MA). Separation was performed using a (30 m x 0.25 mm x 0.25 μm) Restek™ Rxi™- 5ms capillary column (Restek Corporation, Bellefonte, PA), using helium as the carrier gas. Samples were dissolved in hexane, and then 1 μl of the solution was introduced via an auto-injector, using a split/split-less injection program, maintaining a constant column flow rate of 1 ml/min. The injector temperature was set to 220°C. The split-less injection sampling was set for 1 min before the split flow was started at 100 ml/min for 1 min. Then a split flow of 50 ml/min was used for the remainder of the program. The initial oven temperature of 60°C was maintained for 1 min, then ramped at 4°C/min to 250°C, with a final ramp to 300°C at 20°C/min, and held there for 10 min. Ions generated by an electron impact (EI) ionization source (70 eV) were introduced into the mass analyzer after a 5-min solvent delay. Centroid mass spectra were acquired in the positive mode, scanning the range *m/z* 50–500, taking 500 ms/scan. An internal standard of permethylated *myo*-inositol was added to all samples to verify retention time repeatability. Four spectra were averaged, and background was subtracted. The retention times and EI spectra of the released and permethylated glycans were compared to those of deutero-reduced, permethylated monosaccharide standards. Data analysis was performed using the software MS Data Review 8.0 (Bruker). Retention times were compared using extracted ion chromatograms (XIC), for the ion signal at *m/z* 101, an ion common to GalNAc and GlcNAc. The EI spectra recorded for the standards and β-elimination products were compared at the same time points.

## Results

### The vast majority of peptides with *O*-HexNAc derive from Gp40, Gp15, and Gp900, which are vaccine candidates

**Fig 1:**
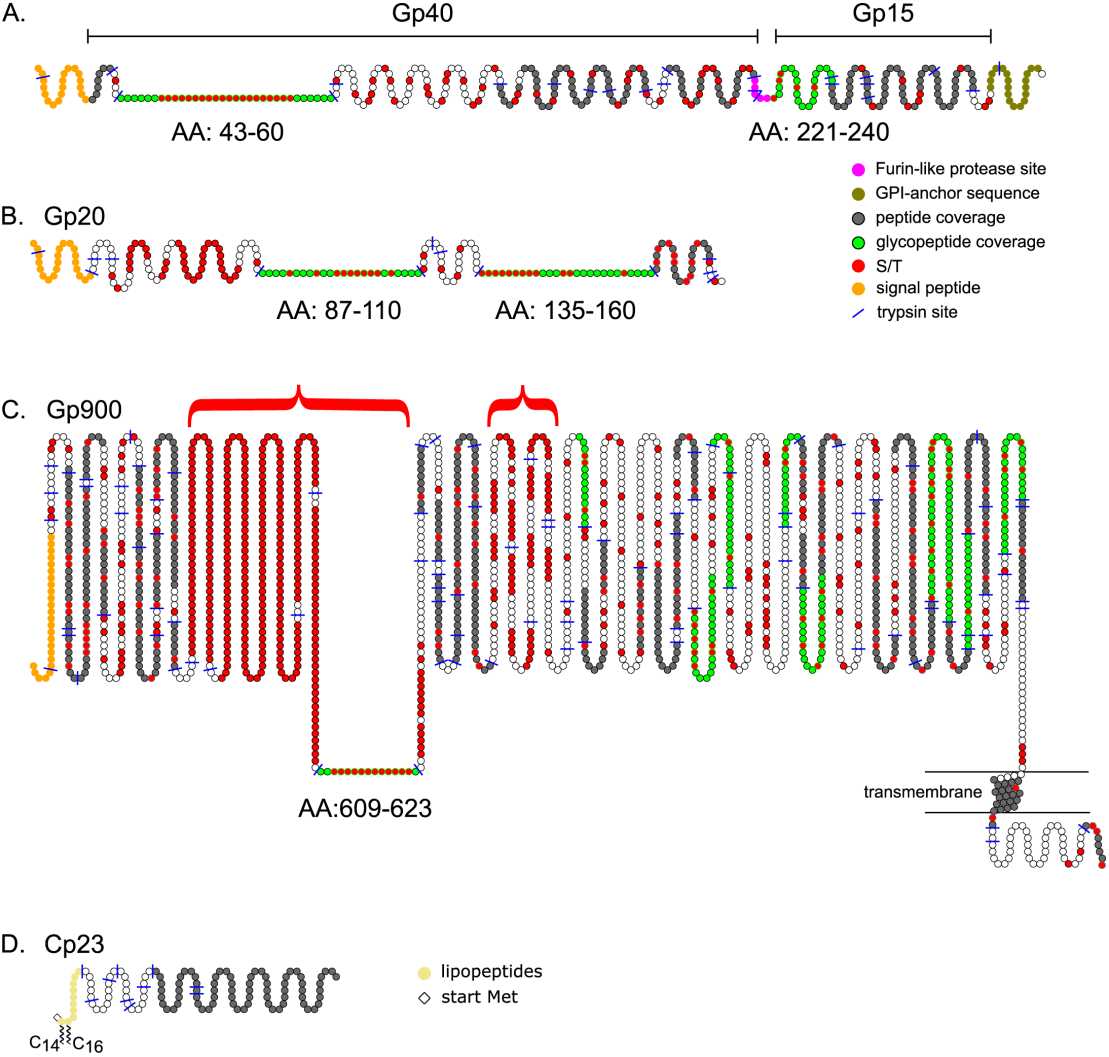
Schematics of *Cryptosporidium* glycoproteins characterized here by mass spectrometry. (A) Gp40/Gp15 precursor is cleaved at a furin-like protease site (pink) into Gp40 and Gp15. Mass spectrometry showed Gp40 has a Thr-rich domain (AA-43 to 60) with numerous *O*-linked HexNAc modifications (marked in green, with Ser and Thr residues marked in red). Gp15 contains a single domain (AA-221 to 240) that is glycosylated. Other peptides identified with mass spectrometry are marked in grey. Predicted N-terminal signal peptide is marked in orange, while GPI-anchor signal is marked in olive. (B) A 20-kDa glycoprotein (Gp20) contains two Thr-rich domains (AA-87 to110 and AA-135 to 160), which contain numerous HexNAc modifications. (C) Gp900 contains two very large Thr-rich domains (red brackets), one of which contains a peptide with three HexNAc residues (AA-609 to 623). The transmembrane helix near the C-terminus is encompassed by two horizontal lines, representing a membrane. (D) The N-terminus of Cp23 is modified with *N-*myristate (C_14_) and *S-*palmitate (C_16_). The start Met is absent (diamond).

Peptides obtained from trypsin digestion of total proteins of *C*. *parvum* oocysts were separated using a UPLC reversed phase C18 column that was online with a mass spectrometer. Peptides were subjected to Higher-energy C-trap Dissociation (HCD), and *O*-glycosylated peptides were recognized by the observation of a sugar-oxonium ion signal at *m/z* 204.0866 in the MS/MS spectra, corresponding to the fragmentation of a precursor containing a HexNAc residue. We reported previously the detection of larger sugar-oxonium ions (corresponding to Hex-HexNAc, HexNAc-HexNAc, and Hex-HexNAc-HexNAc) that all derive from *N*-glycans [54]. Since there was no enrichment for glycoproteins in the protein preparations (*e*.*g*. lectin chromatography), we identified the most abundant glycopeptides without selection bias. Glycopeptides with *O*-linked glycans originated from three *C*. *parvum* vaccine candidates (Gp15, Gp40, and Gp900), as well as Gp20, the immunogenicity of which is unknown (Fig 1, Table 1, and S1 Excel File). We also detected the presence of myristate and palmitate on an N-terminal peptide of the immunodominant antigen Cp23. In addition, we identified at least two peptides without *O*-HexNAc from each of 811 other *C*. *parvum* proteins. Information about these peptides and the glycopeptides described below has been deposited in the ProteomeXchange Consortium.

**Table 1:** Overview of tryptic glycopeptides identified by mass spectrometry.

Protein	Peptide^†^	#HexNAc
Gp40	_(31)_DVPVEGSSSSSSSSSSSSSSSSSTSTVAPANK_(62)_	15–20*
Gp15	_(221)_ETSEAAATVDLFAFTLDGGK_(240)_	1,2, 3*,4
Gp20	_(87)_EGEETDENTDETTTTTTTASPKPK_(110)_	6,7*,8
	_(135)_SSTTTTTTTAPVSSEDNKPEDSEDEK_(160)_	8
Gp900	_(609)_KPTTTTTTTTTTTTK_(623)_	3
	_(958)_IADTSNLFPVQTHK_(971)_	1
	_(1197)_TPTQTDSVTGKPIDPTTGLPFNPPTGH_(1223)_	1
	_(1197)_TPTQTDSVTGK_(1207)_	1
	_(1243)_YAVSNGIKTDNVYGLPVDEITGLPK_(1267)_	1
	_(1248)_NGIKTDNVYGLPVDEITGLPK_(1267)_	1
	_(1373)_GKDGLIVPPTNSINK_(1387)_	1
	_(1410)_VIPGSLPGSLNYPSFNTPQQTDEITGK_(1436)_	1
	_(1646)_TIPGSAASVIHTALGTPTQTDPTTGLPSDPSTGLPFIPGFNVLVDPQTGEQIK_(1698)_	1
	_(1658)_ALGTPTQTDPTTGLPSDPSTGLPFIPGFNVLVDPQTGEQIK_(1698)_	1
	_(1710)_EKNIVTEAAYGLPVDPK_(1726)_	1
	_(1795)_LIDPESGIAIDNSVSGVFATVPGTAAPK_(1822)_	1
	_(1813)_ATVPGTAAPK_(1822)_	1

The number of HexNAc residues is listed for each glycopeptide, and the most abundant glycopeptide in each set is marked with an asterisk. Missed and non-tryptic cleavages are omitted, unless these occurrences led to a change in the number of Ser or Thr residues.

^†^The complete list of glycopeptides used to generate this table can be viewed in S1 Excel File.

### Dense arrays of *O*-GalNAc are present on the Ser-rich domain of Gp40

**Fig 2:**
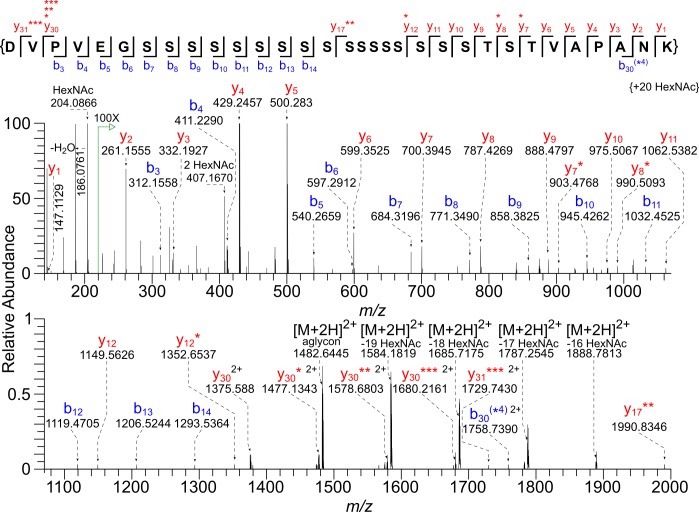
HCD MS/MS spectrum (@ 30 V) of a tryptic glycopeptide of Gp40 shows saturation of an array of Ser residues with *O*-HexNAc residues. The monoisotopic mass of the precursor ion [M + 4H]^4+^ *m/z* 1757.2272 corresponds to the value calculated for the peptide _(43)_DVPVEGSSSSSSSSSSSSSSSSSTSTVAPANK_(60)_ with the addition of 20 HexNAc residues (Δ 0.2 ppm). A very abundant HexNAc oxonium ion (*m/z* 204.0866) and a very low abundance peak (0.5%) corresponding to a HexNAc dimer (*m/z* 407.1670) are present. Asterisks mark the number of HexNAc residues present on b and y ions. Please see S1 Fig for a 45-V HCD MS/MS spectrum of the same Gp40 glycopeptide. The lists of the b and y ions assigned for MS/MS spectra shown in this figure and others can be found in S2 Excel File.

Gp40/Gp15 precursor (cgd6_1080) has an N-terminal signal peptide, a furin cleavage site that separates Gp40 (AA-22 to 220) from Gp15 (AA-221 to 324), and a C-terminal site for the addition of a GPI-anchor (Fig 1) [18–21]. A tryptic glycopeptide (AA-43 to 60) of Gp40, which contains 17 consecutive Ser residues followed by Thr-Ser-Thr, was found to be modified with 15 to 20 HexNAc residues (Table 1 and S1 Excel File). For example, the monoisotopic mass of the precursor ion *m/z* 1757.2272 [M + 4H]^4+^ corresponds to the value calculated for the peptide _(43)_DVPVEGSSSSSSSSSSSSSSSSSTSTVAPANK_(60)_ with the addition of 20 HexNAc residues (Fig 2 and S2 Excel File). The very abundant HexNAc oxonium ion (*m/z* 204.0866) and a very low abundance peak that fits the value for HexNAc_2_ (*m/z* 407.1670) are present in the 30-V HCD MS/MS spectrum. The observed dimer could be an artifact generated in the gas phase from the high population of HexNAc monomers. To a very large extent, glycan loss occurs prior to fragmentation of the peptide, with the result that the observed b and y ions contain zero to four HexNAc residues. The only product ion that can be used to assign the HexNAc modification to a specific amino acid is the y_7_* ion, indicating the presence of HexNAc on the Thr closest to the C-terminus. In a second experiment, to avoid overpopulating the orbitrap analyzer with the less informative HexNAc oxonium ion, the start of the selection window was raised from *m/z* 100 to *m/z* 210, and, to ensure the generation of more peptide backbone fragments, the HCD energy was increased to 45 V (S1 Fig and S2 Excel File). The 45-V HCD MS/MS spectrum exhibited extensive fragmentation of the aglycon peptide, which resulted in product ions that composed a nearly complete b and y series (y2—y_25,_ y_30_) and (b_2_—b_4,_ b_6_—b_7_) (S1 Fig).

Because we saw little evidence for the presence of HexNAc-HexNAc, we assume that each of the 20 potential *O*-glycan sites is occupied with a single HexNAc residue. We were unable to localize site occupancy in glycopeptides with 15–19 HexNAc residues, due to the labile nature of the *O*-glycans. Quite likely, the peptides modified with 15 to 19 HexNAc residues are a mixture of components having different occupancies.

Release of *O*-glycans from *C*. *parvum* sporulated oocyst proteins by reductive β–elimination, followed by GC/MS monosaccharide analysis versus sugar standards, showed that the HexNAc residues in the Gp40 glycopeptide and in glycopeptides of the other vaccine candidates are likely GalNAc (S2 Fig). However, we cannot rule out a small amount of GlcNAc, which was suggested by Western blots of Gp15 [71]. In support of this assignment are the previous reports that *C*. *parvum* has four *O*-GalNAcTs, and patient sera recognize synthetic glycopeptides derived from Gp40 and Gp15 with *O*-GalNAc [32, 33]. In summary, the Gp40 spectra presented here show that the *C*. *parvum O*-GalNAcTs are capable of saturating or nearly saturating consecutive arrays of Ser residues.

### Isolated *O*-GalNAc residues decorate a glycopeptide of Gp15

**Fig 3:**
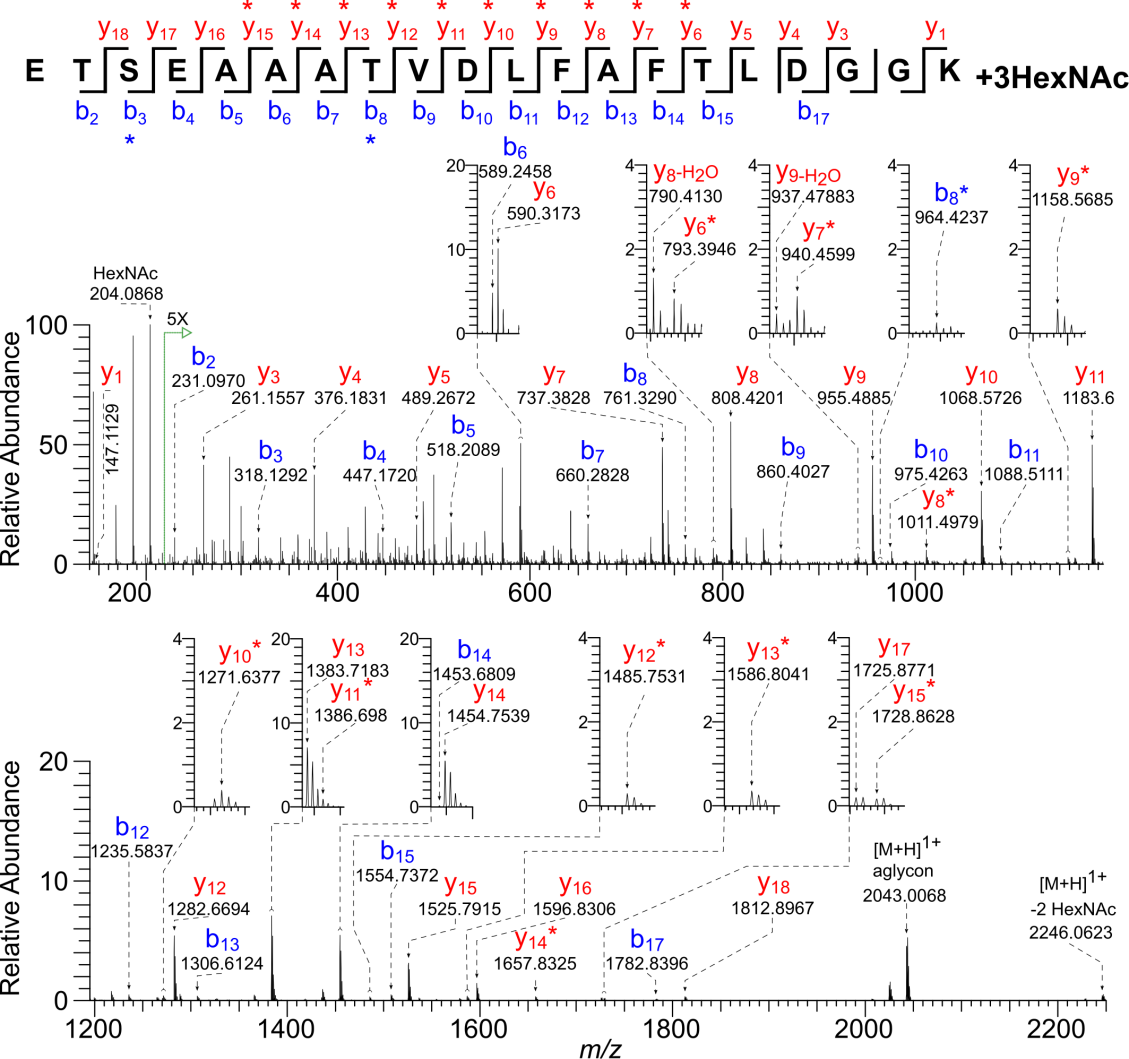
HCD MS/MS spectrum (@ 30 V) of a tryptic glycopeptide of Gp15 shows that three of four *O*-glycan sites contain the HexNAc modification. The precursor ion [M + 2H]^2+^ *m/z* 1326.6164 of the most abundant Gp15 glycopeptide has a monoisotopic mass corresponding to that calculated for the peptide _(221)_ETSEAAATVDLFAFTLDGGK_(240)_ with the addition of three HexNAc residues (Δ 0.3 ppm). There is a prominent HexNAc oxonium ion (*m/z* 204.0868) and full series of b and y ions, some of which contain a single HexNAc residue (*).

A non-tryptic glycopeptide of Gp15 (AA-221 to 240), which results from cleavage of the Gp40/Gp15 precursor by the furin-like protease, contained one to four HexNAc modifications (Fig 1, Table 1, and S1 Excel File) [18–21]. For example, the precursor ion *m/z* 1326.6164 [M + 2H]^2+^ of the most abundant Gp15 glycopeptide has a monoisotopic mass equal to that of the peptide _(221)_ETSEAAATVDLFAFTLDGGK_(240)_ with the addition of three HexNAc residues (Fig 3 and S2 Excel File). Fragmentation with 30-V HCD yielded a prominent HexNAc oxonium ion (*m/z* 204.0868) and full series of b and y ions, some of which retained a single HexNAc modification (marked with an asterisk). The product ion series y_6_* to y_12_* indicates Thr-235 is modified, and the series y_13_* to y_15_* suggests that either Thr-228 or Thr-235 is modified. The b_3_* ion indicates that either Thr-222 or Ser-223 is modified. Thus there is evidence for distribution of the three HexNAc residues over the four available sites in this peptide. In the glycopeptide with four HexNAc modifications, all possible *O*-glycan sites are occupied. Analyses of the fragmentation patterns of numerous other peptides (S1 Excel File), both tryptic and non-tryptic, suggest that Thr-222 is preferentially modified over Ser-223, while Thr-228 and Thr-235 are nearly always modified.

### Dense arrays of *O*-GalNAc are present on Thr-rich glycopeptides of Gp20

**Fig 4:**
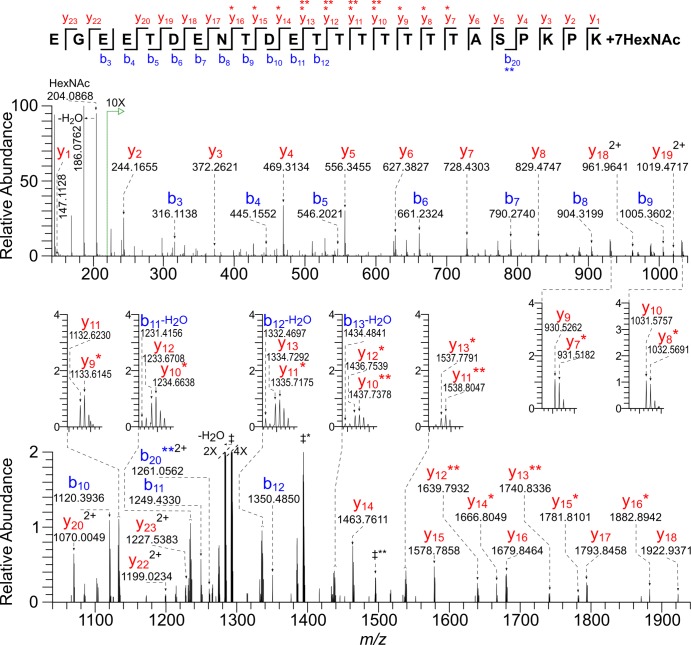
HCD MS/MS spectrum (@ 30V) of a tryptic glycopeptide of Gp20 shows near saturation of consecutive Thr residues with the *O*-HexNAc modification. The precursor ion [M + 4H]^4+^ *m/z* 1001.9305 has a monoisotopic mass corresponding to that calculated for the peptide _(87)_EGEETDENTDETTTTTTTASPKPK_(110)_ plus seven HexNAc residues (Δ 0.5 ppm). There is a prominent HexNAc oxonium ion (*m/z* 204.0868) and full series of b and y ions, some of which contain one (*) or two (**) HexNAc residues. All ions are singly charged, except where indicated. In addition, charge-reduced ions, all 2+, which correspond to species that have undergone consecutive losses of HexNAc residues, are labeled as follows: ‡** = [M + 2H]^2+^—HexNAc_5_ (*m/z* 1495.1470), ‡* = [M + 2H]^2+^—HexNAc_6_ (*m/z* 1393.6224), ‡ = [M + 2H]^2+^ aglycon peptide (*m/z* 1292.0851).

Gp20 (cgd7_1280), is a small, acidic, secreted protein with four domains with consecutive Thr residues, two of which are described here (Fig 1). The first Gp20 glycopeptide _(87)_EGEETDENTDETTTTTTTASPKPK_(110)_ has 10 potential *O*-glycan sites and was found to be decorated with six to eight HexNAc residues (Table 1 and S1 Excel File). For example, the peak corresponding to the precursor ion of the most abundant Gp20 glycopeptide has a monoisotopic [M + 4H]^4+^ *m/z* 1001.9305 equal to the value calculated for the peptide modified by seven HexNAc residues (Fig 4 and S2 Excel File). The 30-V HCD MS/MS spectrum includes a HexNAc oxonium ion (*m/z* 204.0868) and numerous b and y ions retaining zero to two HexNAc residues (marked with asterisks). Because the vast majority of HexNAc residues were lost prior to peptide fragmentation, it was not possible to define the seven occupied sites or to determine whether the occupancy was heterogeneous. A second Gp20 glycopeptide _(135)_SSTTTTTTTAPVSSEDNKPEDSEDEK_(160)_ with 12 potential *O*-glycan sites has a monoisotopic mass equal to that of the peptide with the addition of eight HexNAc residues (Table 1 and S1 Excel File). Again glycan loss prior to peptide backbone fragmentation made it impossible to localize the occupied *O*-glycans sites. Two other Thr-rich domains of Gp20 are present in a 55-amino acid tryptic peptide that was not identified. Regardless, the two Gp20 spectra show that the *C*. *parvum O*-GalNAcTs are capable of nearly saturating arrays of Thr residues.

### A glycopeptide of Gp900 with consecutive Thr residues is lightly modified by *O*-GalNAc, while numerous Gp900 glycopeptides contain a single *O*-HexNAc residue

Gp900 (cgd7_4020), which has an N-terminal signal peptide and a transmembrane domain near its C-terminus, is by far the largest of the *C*. *parvum* vaccine candidates (1912 amino acids minus the signal peptide) (Fig 1) [16, 17]. One reason for the large size of Gp900 is the presence of a vast array of consecutive Thr residues, which extends from AA-304 to 640. A second Thr-rich region extends from AA-797 to 908. Because of the paucity of tryptic sites in the Thr-rich arrays of Gp900, and the likelihood that the Thr stretches are also heavily *O*-glycosylated, these regions were not observed by mass spectrometry, with one exception (Table 1, S1 Excel File, and S3 Fig). The precursor ion *m/z* 732.0284 [M + 3H]^3+^ has a monoisotopic mass equal to that calculated for the peptide _(609)_KPTTTTTTTTTTTTK_(623)_ with the addition of only three HexNAc residues, despite the presence of 12 available sites (S3 Fig and S2 Excel File). The 30-V HCD MS/MS spectrum includes a HexNAc oxonium ion (*m/z* 204.0868) and numerous b and y ions containing zero to two HexNAc residues (marked with asterisks). Here again, because of the lability of the glycans, it was not possible to precisely define the occupied sites or to determine whether the occupancy was heterogeneous.

**Fig 5:**
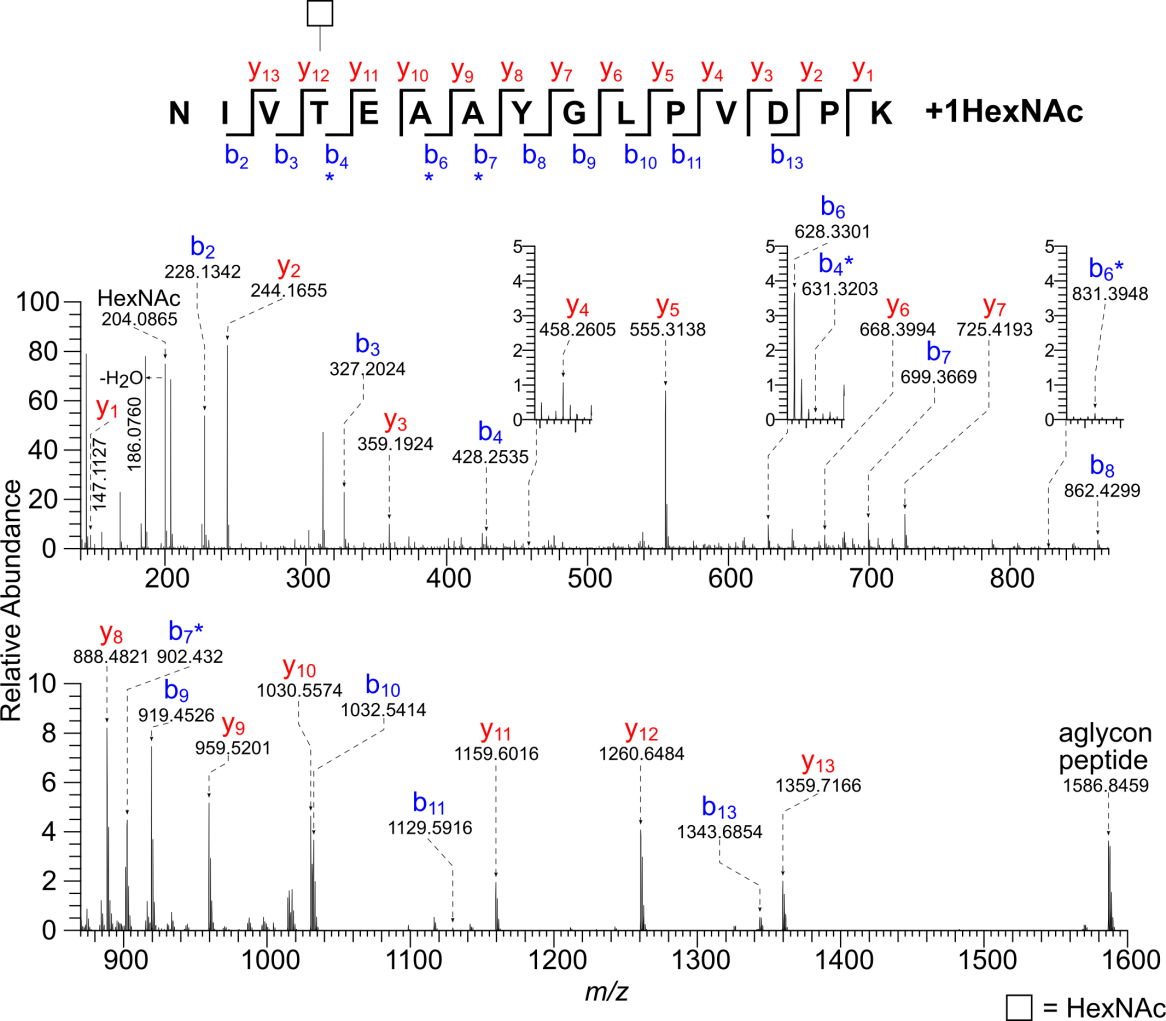
HCD MS/MS spectrum (@ 30V) of a tryptic glycopeptide of Gp900 shows a single *O*-HexNAc modification on a Thr residue. The precursor ion [M + 4H]^4+^ *m/z* 895.4646 has a monoisotopic mass corresponding to that calculated for the peptide (1712)NIVTEAAYGLPVDPK(1726) plus a single HexNAc residue (Δ 0.1 ppm). There is a prominent HexNAc oxonium ion (*m/z* 204.0865) and full series of b and y ions, some of which contain a HexNAc residue (*). The b_4_*, b_6_*, and b_7_* ions show that Thr-1715 is modified.

Many of the most abundant glycopeptides of Gp900 have a single HexNAc modification at an isolated Ser or Thr residue (Table 1 and S1 Excel File). For example, the precursor ion *m/z* 895.4646 [M + 4H]^4+^ has a monoisotopic mass corresponding to the value calculated for the peptide (1712)NIVTEAAYGLPVDPK(1726) plus a single HexNAc residue (Fig 5 and S2 Excel File). The b_4_*, b_6_*, and b_7_* ions show that Thr-1715 is modified. The mass spectra of 12 unique peptides from Gp900, each with a single HexNAc modification, together with the spectra from Gp15, suggest that the *C*. *parvum O*-GalNAcTs are capable of modifying isolated Ser and Thr residues, in addition to stretches of consecutive Ser residues in Gp40 and Thr in Gp20 and Gp900.

### At the N-terminus of Cp23 myristoyl modifies Gly1, while palmitoyl modifies Cys2

**Fig 6:**
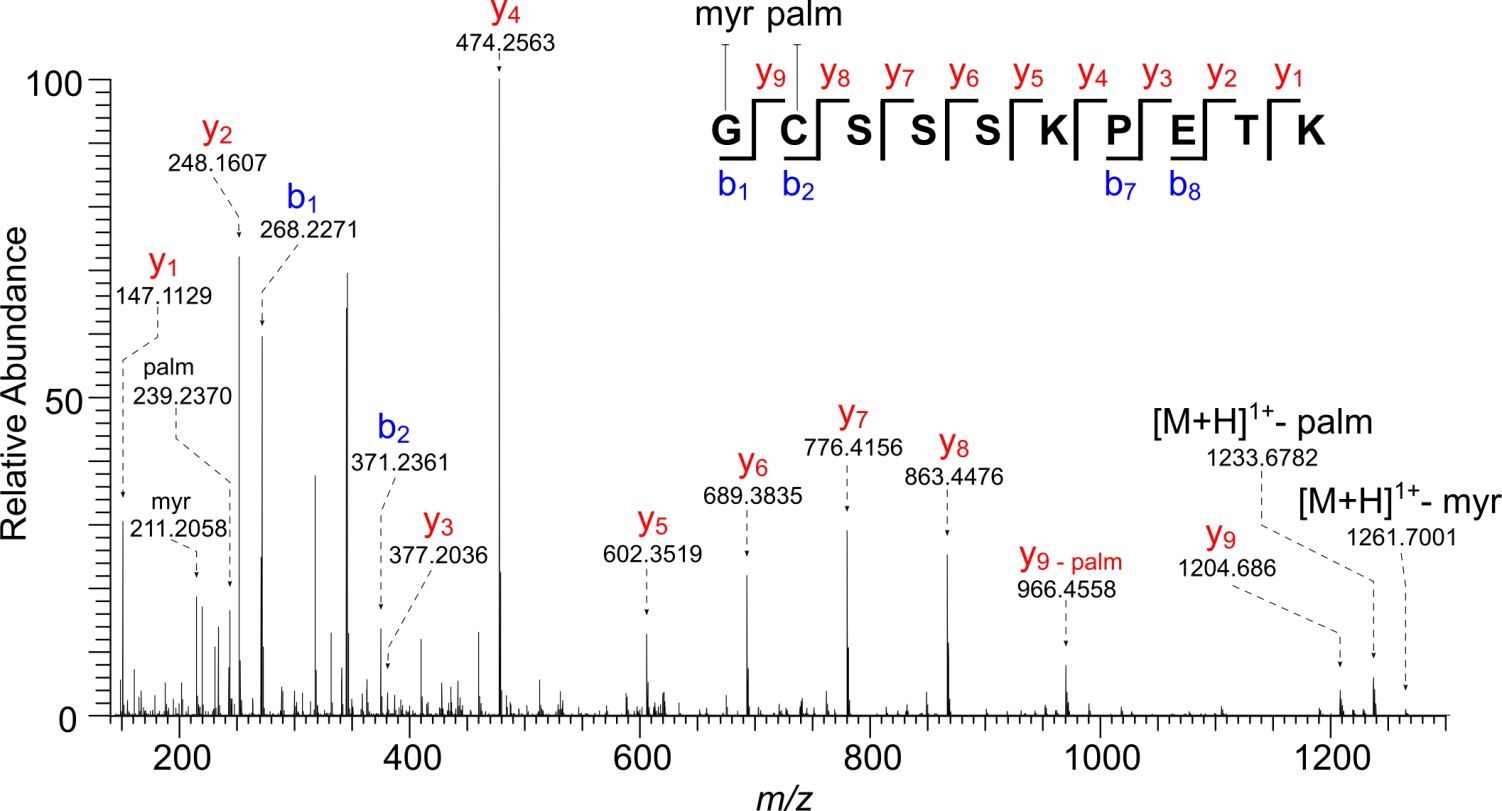
HCD MS/MS spectrum (@30 V) of an N-terminal peptide of Cp23 (minus Met-1) shows Gly-1 is modified with myristate and Cys-2 is modified with palmitate. The precursor ion [M + 2H]^2+^ *m/z* 736.4573 has a monoisotopic mass corresponding to that calculated for the peptide _(2)_GCSSSKPETK_(11)_ plus myristate and palmitate (Δ 1.1 ppm). Fragment ions could be assigned to myristate (*m/z* 211.2056) and palmitate (*m/z* 239.2370), as well as the charge-reduced [M + H]^1+^ molecular ions that have undergone loss of palmitate (*m/z* 1233.6782) or myristate (*m/z* 1261.7001). All the appropriate b/y ions contain the lipid modification, unless otherwise indicated.

The immunodominant antigen Cp23 (cgd4_3620) contains no signal peptide but has an N-terminal sequence (2)GCSSSKPETK(11) similar to those modified by fatty acyl chains in the host and other apicomplexans (Fig 1) [52, 72–78]. Consistent with this resemblance, numerous hydrophobic peptides were identified by mass spectrometry containing the N-terminus of Cp23 minus Met-1, with no modification, substituted by either myristate or palmitate, or both (S1 Table.) [56]. For example, the precursor ion [M + 2H]^2+^ *m/z* 736.4573 has a monoisotopic mass equal to that calculated for the peptide GCSSSKPETK with the addition myristate and palmitate (Fig 6 and S2 Excel File). Fragmentation using 30-V HCD showed the presence of myristate (*m/z* 211.2056) and palmitate (*m/z* 239.2370), as well as the charge reduced [M + H]^1+^ molecular ion with loss of palmitate (*m/z* 1233.6782) or myristate (*m/z* 1261.7001). The presence of a complete y -ion series and a partial b -ion series allowed us to assign myristate to the N-terminal Gly and palmitate to the Cys. We believe the example given is what is present on the native protein. The peptides where palmitate is absent and Cys is carbamidomethylated or palmitate modifies Ser residues arose during sample processing [56, 57].

## Discussion

Mass spectrometry here directly demonstrated that addition of *O*-linked HexNAc (presumably *O*-GalNAc) is a widespread modification of *C*. *parvum* vaccine candidates (Gp15, Gp40, and Gp900) [11, 12]. Previous evidence for the addition of *O*-GalNAc to these proteins has been obtained through the use of synthetic glycopeptides, lectins, patient sera, or a monoclonal anti-carbohydrate antibody to *C*. *parvum* [25, 33]. *O*-GalNAc modifications saturate Ser-rich sequences of Gp40, and they nearly saturate Thr-rich sequences of Gp20, a previously uncharacterized protein. Nearly all of peptides with *O*-glycans derive from just four proteins (Gp40, Gp15, Gp900, and Gp20), even though >800 proteins were identified by mass spectrometry. Limitations of our observations include 1) failure to observe most of the Thr-rich domains of Gp900 and two of the Thr-rich domains of Gp20, 2) inability to assign *O*-glycans sites on many of the peptides due to loss of the *O*-linked glycan residues during HCD fragmentation, and 3) limited sampling of glycoproteins with *O-G*alNAc. In particular, the GalNAc-binding *Maclura pomifera* agglutinin previously enriched six mucin-like glycoproteins in addition to Gp40, Gp15, and Gp900 from lysed oocysts [15]. Other glycoproteins with *O*-GalNAc beyond those described here and the six mucin-like glycoproteins are certainly likely.

These results show that the four *O*-GalNAcTs of *C*. *parvum*, each of which has a lectin domain in addition to its glycosyltransferase domain, efficiently continue to glycosylate regions of glycoproteins that are already glycosylated [32, 79, 80]. Indeed the four *O*-GalNAcTs of *C*. *parvum* are able to make the same kind of modifications to arrays of Ser and Thr and to isolated Ser and Thr as the 20 *O*-GalNAcTs of the host. In studies beyond the scope of those performed here, the activity of each *O*-GalNAcT might be determined by 1) recombinant expression of enzymes with peptide substrates or 2) knockouts of the genes encoding these enzymes, a technology that is now available in *C*. *parvum* grown in mice [81]. *O*-glycans of *C*. *parvum* differ from those of the host in that *O*-GalNAc is not extended by other sugars [80]. *C*. *parvum* then is the equivalent of the “SimpleCell” lines engineered to express truncated *O*-GalNAc (knockout of cosmc gene), which have been used to map occupied *O*-glycan sites [82–84]. Similarly, the unextended *C*. *parvum O*-glycans are recognized by anti-Tn antibodies, which bind to unextended *O*-GalNAc [33, 85].

Properties that distinguish *C*. *parvum* Gp15 and Gp40 include glycosylation (discrete *O*-GalNAc residues versus densely clustered *O*-GalNAc residues), localization on sporozoites (apically associated versus diffusely covering surface), localization on oocyst walls (outer surface versus inner surface), and structure (GPI-anchored versus secreted) [15–23, 25]. We infer that the densely clustered *O*-GalNAc residues make the Ser-rich regions of Gp40 and Thr-rich regions of Gp20 and Gp900 rigid and extended rather than unstructured [86–88]. These extended regions of *O*-glycosylation may contribute to the tethering function of Gp40 and Gp900, which attach sporozoites to the inner layer of the oocyst wall [15]. *O*-glycosylation on these glycoproteins that coat the sporozoite surface may also affect host cell invasion and/or the innate and acquired immune responses to infecting parasites [25, 26, 28–30]. For example, the *C*. *parvum* galactose/GalNAc lectin binds to *O*-GalNAc on Gp40 and Gp900 [24]. In contrast, addition of myristate to N-terminal Gly and palmitate to Cys likely directs Cp23 from the cytosol to membranes of *C*. *parvum* and thus is likely important for its function, as has been extensively studied in *Toxoplasma*, *Plasmodium*, and the host [50, 72–78]. Chemical biology experiments or mutation of sites for addition of myristate and palmitate on Cp23 would be useful to test the roles of fatty acyl modifications in *C*. *parvum*. It is not clear how vaccination with recombinant Cp23, which is presumably a cytosolic protein, even if membrane bound, produces a protective immune response.

Serological screens for *C*. *parvum* use recombinant proteins made in bacterial systems that fail to add *O*-GalNAc (Gp40 and Gp15) or fatty acyl chains (Cp23) [26, 28–30, 36–38]. Because the host antibody response includes antibodies to glycopeptides with *O*-glycans including some with *O*-GalNAc on sites described here [33], these serological screens are likely lacking sensitivity and might be improved by expressing *C*. *parvum* proteins in SimpleCells that add unextended *O*-GalNAc to glycoproteins [82–84]. Similarly, vaccination with recombinant proteins produced in bacteria produces an immune response to the unmodified peptides, whereas acquired immunity to *C*. *parvum* infections includes responses to the *O*-glycans on Gp40 and Gp15 [33] and possibly lipid-modifications of Cp23. Again, the cosmc knockout might be used to produce recombinant Gp40 or Gp15 coated with unextended *O*-GalNAc for vaccination. Production of Cp23 in mammalian cells that add fatty acyl chains might increase the sensitivity of serological screens for this antigen and generate a better vaccine.

## Supporting information

S1 Excel File An Excel spreadsheet, summarizing the 358 spectra identified containing HexNAc(s). The list of unique peptides was used to make Table 1 in the main text, as shown as the representative peptides (sheet 1). The Gp40 peptides differ in the number of HexNAc residues present and the number of spectra observed. There are 14 unique peptide sequences with varying number of HexNAc residues, and N-terminal degradation products represented by the Gp15 peptide in Table 1. There are two unique glycopeptide sequences represented by two of the Thr-rich peptides of Gp20. The first peptide has two unique sequences, resulting from an atypical trypsin cleavage between the terminal (107)PKPK(110), with cleavage after Lys-108. Sheet 2 contains detailed information about each manually verified PEAKS spectrum match. These spectra were re-annotated using the GlycoReSoft software developed in-house, as described in the methods section. These re-annotated spectra have been deposited in the PRIDE repository as.svg image files, Their concatenated name refers to the Source File-raw and the Scan# of the individual spectrum (columns O and P, respectively, in sheet 2 of S1 Excel File). An example re-annotated spectrum is provided in S4 Fig.
(XLSX)

S2 Excel File An Excel spreadsheet of peak lists and ion assignments for the mass spectra shown in Figs 2 to 6, S1, and S3. (XLSX)

S1 Fig HCD MS/MS spectrum (@ 45V) of a tryptic glycopeptide of Gp40 gives complete sequence of the peptide. The precursor ion [M + 4H]^4+^ *m/z* 1757.2272 corresponds to the monoisotopic mass equal to that of the peptide (43)DVPVEGSSSSSSSSSSSSSSSSSTSTVAPANK(60) with the addition of 20 HexNAc residues (Δ 0.6 ppm). The selection window was set to start at *m/z* 210 in order to exclude the very abundant oxonium ion (*m/z* 204.0866). There is extensive fragmentation of the aglycon peptide ((y2—y_25,_ y_30_) and (b_2_—b_4,_ b_6_—b_7_) ions). Fig 2 shows the 30-V HCD MS/MS spectrum of the same peptide.
(TIFF)

S2 Fig GC-MS analysis shows the *O*-glycan released by β–elimination from *C*. *parvum* glycoproteins is GalNAc. GC/MS data obtained for the deuteroreduced and permethylated glycan released by reductive β-elimination of *C*. *parvum* oocyst glycoproteins are compared with results observed for the GlcNAc and GalNAc standards, which were similarly treated. Extracted ion chromatograms of *m/z* 101 are shown on the left. Electron impact mass spectra of the standards and the product from reductive β-elimination of *C*. *parvum* oocyst glycoproteins are shown on the right. The sugar released from *C*. *parvum* is assigned as GalNAc, because the retention time (34 min) and EI mass spectrum both match those of the standard GalNAc. The EI mass spectra of the components eluting at positions marked with one asterisk (*) and two asterisks (**) do not correspond to sugar derivatives.
(TIFF)

S3 Fig HCD MS/MS spectrum (@ 30V) of a tryptic glycopeptide of Gp900 shows partial glycosylation of a Thr-rich repeat. The precursor ion [M + 3H]^3+^ *m/z* 732.0284 has a monoisotopic mass corresponding to the peptide (609)KPTTTTTTTTTTTTK(623) with the addition of three HexNAc residues (Δ -0.1 ppm). There is a prominent HexNAc oxonium ion (*m/z* 204.0868) and a full series of b and y ions, some of which contain one (*) or two (**) HexNAc residues. The loss of HexNAc residues made it impossible to localize occupied sites. Charge-reduced aglycon peptide ions are observed in the spectrum: ǂ = [M + 2H]^2+^ *m/z* 792.9193 and † = [M + H]^1+^ *m/z* 1584.8348.
(TIFF)

S4 Fig A representative GlycReSoft re-annotated spectrum, one of 345 tandem mass spectra deposited into the PRIDE repository. This figure presents an example of an MS/MS spectrum initially assigned by PEAKS DB, then manually verified and re-annotated using the in-house software GlycReSoft. GlycReSoft is capable of discovery and annotation, but only the annotation capabilities were utilized here.
(TIFF)

S1 Table Lipid modifications of the N-terminus of Cp23. (DOCX)
