# Supplementary figures and images for: Cryptosporidium parvum vaccine candidates are incompletely modified with O-linked-N-acetylgalactosamine or contain N-terminal N-myristate and S-palmitate
Source: PLoS One. 2017 Aug 8;12(8):e0182395. doi: 10.1371/journal.pone.0182395 (PMC5549699; doi:10.1371/journal.pone.0182395)

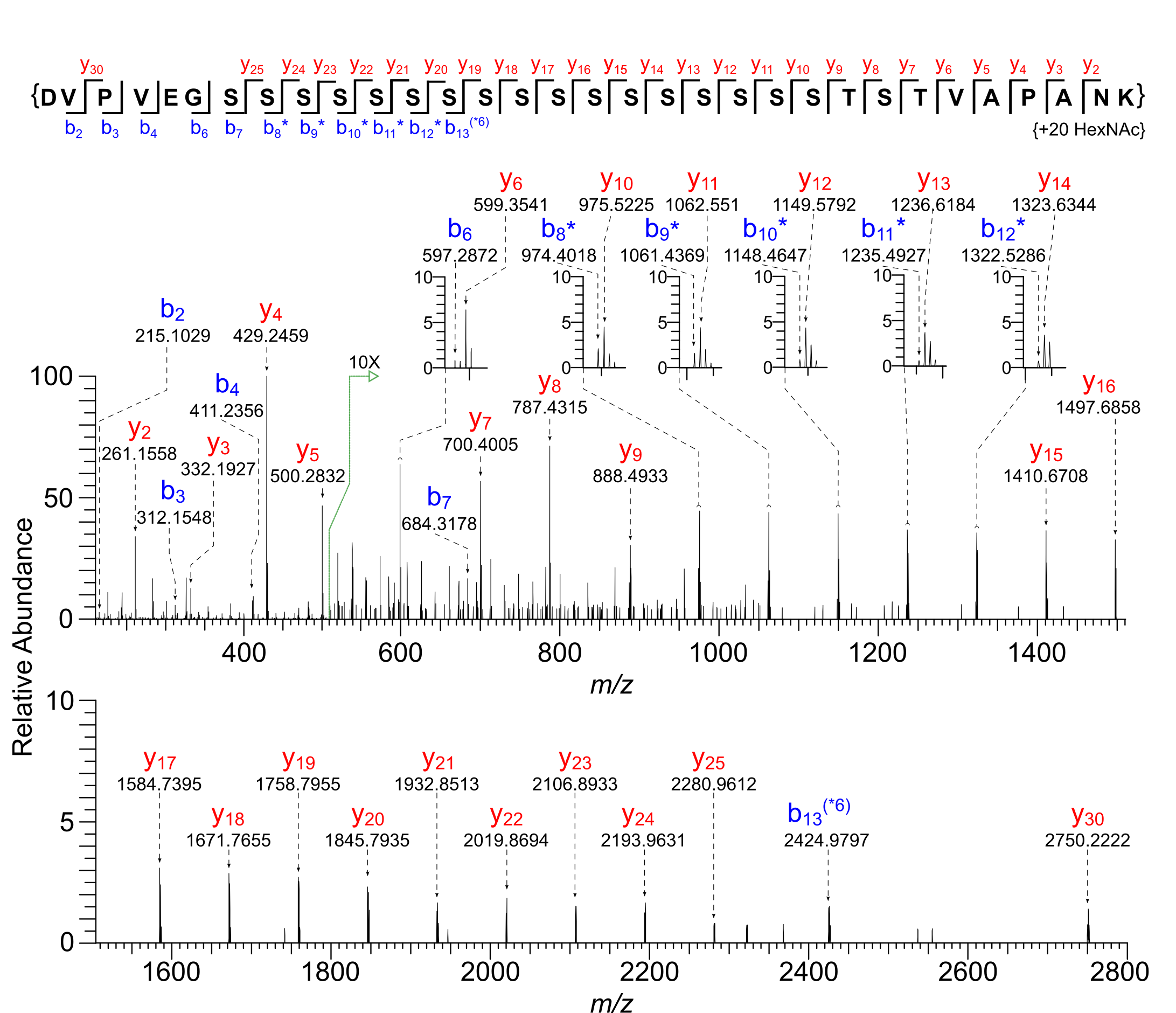

Supplement: S1 Fig — The precursor ion [M + 4H]4+ m/z 1757.2272 corresponds to the monoisotopic mass equal to that of the peptide (43)DVPVEGSSSSSSSSSSSSSSSSSTSTVAPANK(60) with the addition of 20 HexNAc residues (Δ 0.6 ppm). The selection window was set to start at m/z 210 in order to exclude the very abundant oxonium ion (m/z 204.0866). There is extensive fragmentation of the aglycon peptide ((y2—y25, y30) and (b2—b4, b6—b7) ions). Fig 2 shows the 30-V HCD MS/MS spectrum of the same peptide. (TIFF) [file pone.0182395.s003.tiff]

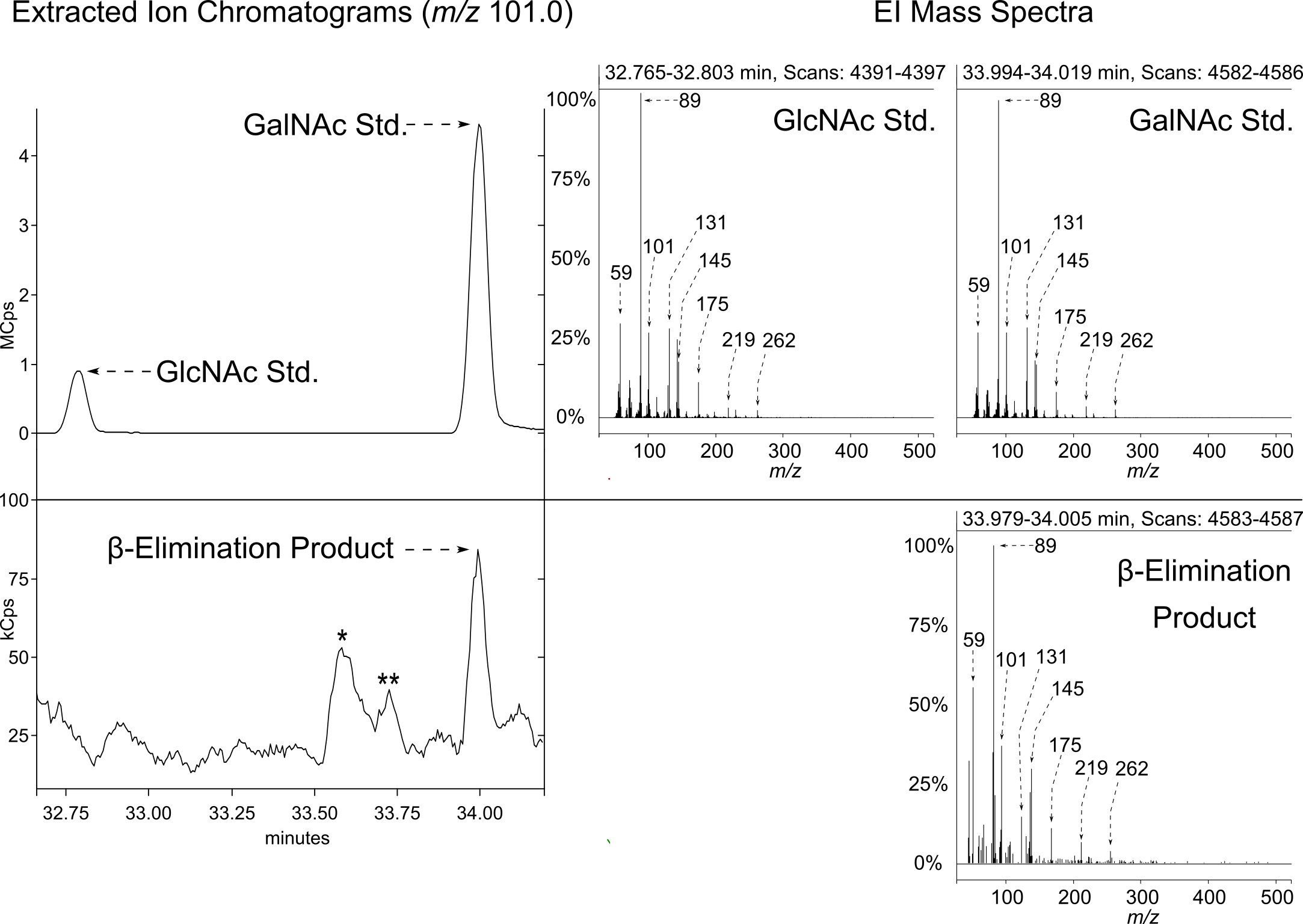

Supplement: S2 Fig — GC/MS data obtained for the deuteroreduced and permethylated glycan released by reductive β-elimination of C. parvum oocyst glycoproteins are compared with results observed for the GlcNAc and GalNAc standards, which were similarly treated. Extracted ion chromatograms of m/z 101 are shown on the left. Electron impact mass spectra of the standards and the product from reductive β-elimination of C. parvum oocyst glycoproteins are shown on the right. The sugar released from C. parvum is assigned as GalNAc, because the retention time (34 min) and EI mass spectrum both match those of the standard GalNAc. The EI mass spectra of the components eluting at positions marked with one asterisk (*) and two asterisks (**) do not correspond to sugar derivatives. (TIFF) [file pone.0182395.s004.tiff]

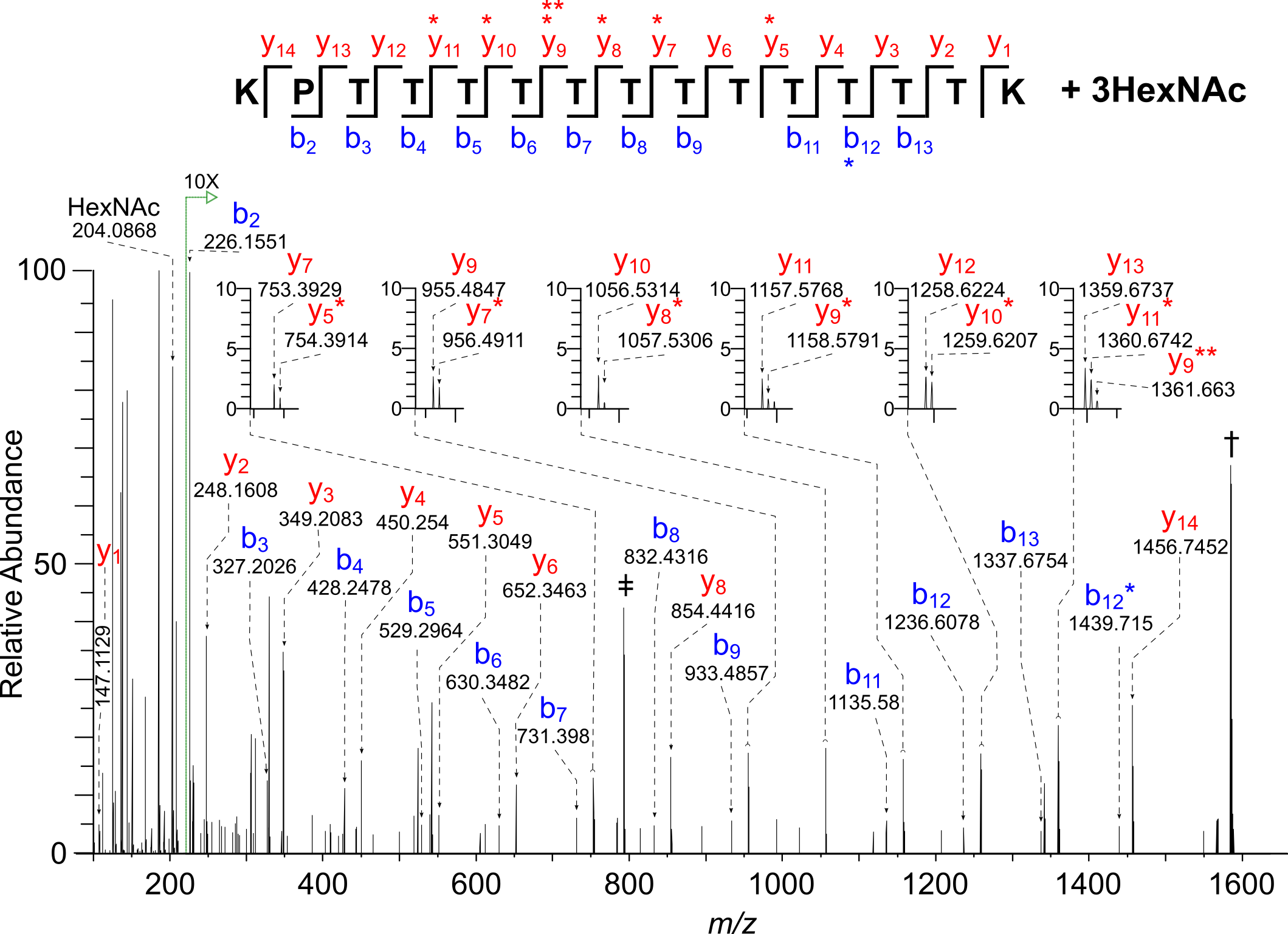

Supplement: S3 Fig — The precursor ion [M + 3H]3+ m/z 732.0284 has a monoisotopic mass corresponding to the peptide (609)KPTTTTTTTTTTTTK(623) with the addition of three HexNAc residues (Δ -0.1 ppm). There is a prominent HexNAc oxonium ion (m/z 204.0868) and a full series of b and y ions, some of which contain one (*) or two (**) HexNAc residues. The loss of HexNAc residues made it impossible to localize occupied sites. Charge-reduced aglycon peptide ions are observed in the spectrum: ǂ = [M + 2H]2+ m/z 792.9193 and † = [M + H]1+ m/z 1584.8348. (TIFF) [file pone.0182395.s005.tiff]

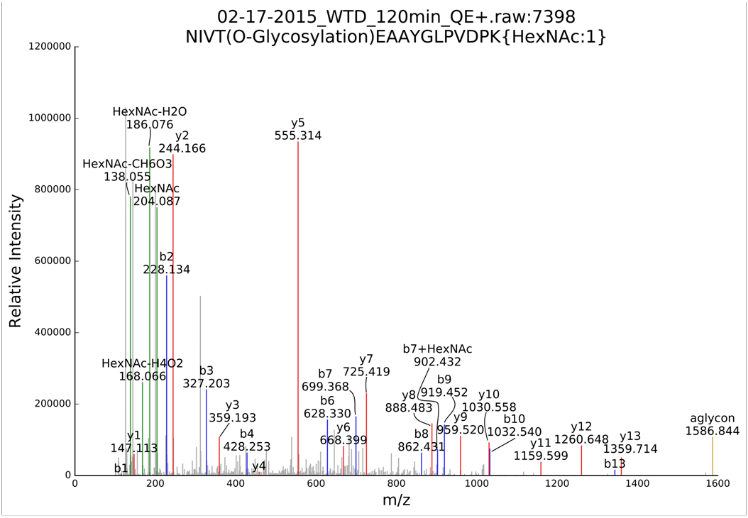

Supplement: S4 Fig — This figure presents an example of an MS/MS spectrum initially assigned by PEAKS DB, then manually verified and re-annotated using the in-house software GlycReSoft. GlycReSoft is capable of discovery and annotation, but only the annotation capabilities were utilized here. (TIFF) [file pone.0182395.s006.tiff]
