# Supplementary material for: Cryptosporidium parvum vaccine candidates are incompletely modified with O-linked-N-acetylgalactosamine or contain N-terminal N-myristate and S-palmitate
Source: PLoS One. 2017 Aug 8;12(8):e0182395. doi: 10.1371/journal.pone.0182395 (PMC5549699; doi:10.1371/journal.pone.0182395)
Supplement: S1 Table — (DOCX) [file pone.0182395.s007.docx]

**S1 Table. Lipid modifications of the N-terminus of Cp23.**

| **Protein** | **Peptide** | **#Spectra** |
| --- | --- | --- |
| Cp23 | _(2)_G^{myr}^C^{cm}^SSSKPETK_(11)_ | 16* |
|  | _(2)_G^{myr}^C^{cm}^SSS^{palm}^KPETK_(11)_ | 2 |
|  | _(2)_G^{myr}^C^{cm}^SS^{palm}^SKPETK_(11)_ | 2 |
|  | _(2)_G^{myr}^C^{cm}^SSSK^{palm}^PETK_(11)_ | 2 |
|  | _(2)_G^{myr}^C^{palm}^SSSKPETK_(11)_ | 2* |
|  | _(2)_GC^{cm}^S^{palm}^SSKPETK_(11)_ | 1 |
|  | _(2)_GC^{cm}^S^{palm}^S^{palm}^SKPETK_(11)_ | 2 |

This table shows the number of spectra observed for the different variants of lipopeptides for the protein Cp23. *Asterisks mark peptides that are likely real, the other peptides could be sample preparation artifacts [56, 57]. ^{cm}^ = carbamidomethyl, Δ+57.0214 Da,^{myr}^ = myristate, Δ+210.1983 Da, ^{palm}^ = palmitate, Δ+238.2296 Da.
